# Country data on AMR in India in the context of community-acquired respiratory tract infections: links between antibiotic susceptibility, local and international antibiotic prescribing guidelines, access to medicine and clinical outcome

**DOI:** 10.1093/jac/dkac212

**Published:** 2022-09-06

**Authors:** Didem Torumkuney, Aruna Poojary, Bhaskar Shenoy, Puja Nijhara, Krunal Dalal, Rendani Manenzhe

**Affiliations:** GlaxoSmithKline, 980 Great West Road, Brentford, Middlesex TW8 9GS, UK; Department of Pathology and Microbiology, Breach Candy Hospital Trust, Mumbai, Maharashtra, India; Department of Paediatrics, Manipal Hospitals, Bangalore, Karnataka, India; GlaxoSmithKline, 252, Dr Annie Besant Road, Worli, 400030, Mumbai, India; GlaxoSmithKline, 252, Dr Annie Besant Road, Worli, 400030, Mumbai, India; GlaxoSmithKline, The Campus, Flushing Meadows, 57 Sloane Street, Bryanston, Gauteng, 2021, South Africa

## Abstract

**Background:**

Antimicrobial resistance (AMR) is one of the biggest threats to global public health. Selection of resistant bacteria is driven by inappropriate use of antibiotics, amongst other factors. COVID-19 may have exacerbated AMR due to unnecessary antibiotic prescribing. Country-level knowledge is needed to understand options for action.

**Objectives:**

To review the current situation with respect to AMR in India and initiatives addressing it. Identifying areas where more information is required will provide a call to action to minimize further rises in AMR and to improve patient outcomes.

**Methods:**

National AMR initiatives, antibiotic use and prescribing in India, and availability of susceptibility data, in particular for the key community-acquired respiratory tract infection (CA-RTI) pathogens (*Streptococcus pneumoniae* and *Haemophilus influenzae*) were identified. National and international antibiotic prescribing guidelines for specific CA-RTIs (community-acquired pneumonia, acute otitis media and acute bacterial rhinosinusitis) commonly used locally were also reviewed, plus local antibiotic availability. Insights from a local clinician and clinical microbiologist were sought to contextualize this information.

**Conclusions:**

Many initiatives have been launched since AMR was recognized as a national priority and organizations such as the Indian Academy of Paediatrics and the Global Antibiotic Resistance Partnership have worked to build awareness. The Indian Ministry of Health and Family Welfare published a 5 year national action plan on AMR. However, the burden of infectious disease and consumption of antibiotics in India is high. There have been national surveillance studies generating local data along with international studies such as Survey of Antibiotic Resistance (SOAR) and Antimicrobial Testing Leadership and Surveillance (ATLAS). For common RTIs, clinicians use a range of international and national guidelines. However, a more standardized inclusive approach to developing local guidelines, using up-to-date local surveillance data from community-acquired infections, could make guidelines more locally relevant. This would encourage more appropriate antibiotic prescribing and improve adherence. This would, in turn, potentially limit AMR development and improve patient outcomes.

## Introduction

Antimicrobial resistance (AMR) is one of the biggest threats to public health throughout the world^1^ as described in the introductory paper of this Supplement.^2^ The WHO states that ‘the world urgently needs to change the way it prescribes and uses antibiotics. Even if new medicines are developed, without behaviour change, antibiotic resistance will remain a major threat’.^3^ The first paper in this Supplement included details about the multiple factors which can drive a rise in AMR, along with the global initiatives that are in place to address this threat.^2^ Each country and/or region must also play their part through local initiatives.

In order to identify how AMR can be addressed in India in the future, it is necessary to review what is happening now. In this paper, we present the current situation in India, determined by using published information (from searching PubMed, Google Scholar and the internet) to ascertain any national initiatives to address AMR, antibiotic use and prescribing, and availability of susceptibility data, in particular for the key community-acquired respiratory tract infection (CA-RTI) pathogens *Streptococcus pneumoniae* and *Haemophilus influenzae*. National and international antibiotic prescribing guidelines for CA-RTIs [specifically community-acquired pneumonia (CAP), acute otitis media (AOM), and acute bacterial rhinosinusitis (ABRS)] commonly used by healthcare professionals were also reviewed, along with how these link to local antibiotic availability. Insights from a clinician and from a clinical microbiologist in India were sought to contextualize this information. In addition, we aimed to identify areas where more information is required and present a call to action to improve clinical outcomes for patients and to minimize further rises in AMR within India.

## Action Plan development

In recent years, addressing AMR has been recognized in India as a national priority and a range of strategies has been introduced, including educational and awareness initiatives, infection control guidelines, audit and feedback, and antimicrobial stewardship (AMS).^4^ Organizations such as the Indian Academy of Paediatrics, the Global Antibiotic Resistance Partnership and the Indian Council of Medical Research (ICMR) have worked to build awareness concerning rising AMR amongst professional bodies, the media, and the general public. A key milestone was the Chennai Declaration of 2012, the output from the first ever meeting of medical societies in India which aimed to address AMR by formulating a 5 year strategy to develop a road map for combating the global challenge of AMR from the Indian perspective.^4^

In 2016, the ‘Medicines with the Red Line’ campaign was launched as a way of addressing the increasing misuse of antibiotics by educating and informing patients. All prescription-only antibiotics were marked with a red line on the packaging and patients were informed that any antibiotic marked in this way should only be taken following prescription and advice from their doctor and the complete course should be taken. The aim was to discourage over-the-counter (OTC) purchase of antibiotics and unnecessary antibiotic prescribing through raised awareness.^4^

Following the formulation by the World Health Assembly in 2015 of a Global Action Plan (GAP) for AMR^5^ many countries began to develop their own National Action Plan (NAP) on AMR. In April 2017, in response to this WHO initiative,^5^ the Indian Ministry of Health and Family Welfare published a 5 year NAP (2017–21) on AMR outlining the priorities and implementation strategies for curbing AMR in India.^4,6^ The first five strategic priorities of NAP-AMR are firmly aligned with the GAP on AMR and an additional sixth strategic priority highlights India’s aspirational aim to contain AMR at an international level with other countries and organizations, national disease control programmes and at a sub-national/state level through development of state action plans on AMR.^4,6^

## Burden of disease

The burden of infectious disease in India (population, as of 2022, 1 403 458 058)^7^ is considered to be amongst the highest in the world and includes infections due to multiresistant pathogens.^6^ In 2010, India was the world’s largest consumer of antibiotics in humans.^4^ Antibiotic consumption in India is high due to: easy access to OTC medicines, self-medication based on word of mouth advice or on internet searching, non-availability or non-utilization of clinical microbiology laboratory services for cultures and antibiotic susceptibility testing (AST), variation in the approach of clinicians to antibiotic prescribing, lack of antibiotic de-escalation, regulatory issues and commercial antibiotic promotion.^4,6^ Underuse of antibiotics due to lack of access to good quality and affordable antibiotics is also common in India, leading to significant mortality (particularly in paediatrics). In addition to human healthcare, antibiotics are also used in animal husbandry, fisheries (for treatment and growth promotion), as well as in agricultural sectors. The lack of knowledge among the public is also a matter of concern regarding appropriate use of antibiotics along with poor compliance and self-medication.^6^ Another factor contributing to the crisis of AMR is environmental pollution due to pharmaceutical, livestock and hospital waste.^4,8^

## Surveillance

### National surveillance studies

In India, several systematic national surveillance studies generating local data have been described.^4,6,8^ There are also a small number of studies from individual units or hospitals which have provided information on local AMR. Whilst these local data are extremely useful within a specific hospital or city, they cannot provide a full picture nationally. Therefore, in response and acting on the aims of the NAP, the Indian Council of Medical Research (ICMR) established an AMR surveillance network (ICMR-AMRSN) in tertiary care hospitals across India.^6,9^ Alongside this network, ICMR has developed a fully customizable tool for the collection, management, analysis and reporting of the AMR data. It is a software package and tool for management and analysis of AST and antimicrobial consumption data collected through multiple laboratories across India and has the additional aim of enforcing quality antimicrobial susceptibility testing in laboratories. The National Centre for Disease Control (NCDC) is the focal point for implementation of the programme and has the overall aim of understanding the extent and pattern of AMR and making use of this information when formulating strategies designed to prevent AMR spread.^6^ The Indian NAP also recognizes that, since microbiologists and clinical microbiology laboratories are the cornerstones for surveillance, it is vital to improve the quality of laboratories by ensuring increased availability of trained personnel and adequate infrastructure, reinforced quality controls, and hands-on training support from tertiary care institutions.^4^

### Global surveillance studies

#### SOAR

Several global surveillance studies provide antibiotic susceptibility data in India. Survey of Antibiotic Resistance (SOAR) is a multinational antibiotic surveillance study, ongoing in an expanding range of countries since 2002. The study aims to collect and make available in published, peer-reviewed papers, antibiotic susceptibility data, specifically for *S. pneumoniae* and *H. influenzae*, the most commonly isolated bacterial respiratory pathogens in the community.^10^ Key features of the SOAR study are that the focus is on only these pathogens, and that identification and susceptibility testing are performed in an independent centralized laboratory using standardized methodology (CLSI) allowing for comparisons to be made between countries or regions and for the identification of trends over time.^10^ New SOAR data is analysed based on three different breakpoints: CLSI, EUCAST dose specific, and PK/PD breakpoints.

Clinical breakpoints are cut-off MIC values used to classify microorganisms into the clinical categories susceptible (S), intermediate (I) and resistant (R) to assist in prediction of the clinical success or failure of a specific antibiotic.^10^ Two main international organizations define breakpoint values: CLSI and EUCAST. Due to variation in criteria for their definition, there are some differences between CLSI and EUCAST in the clinical breakpoint values for certain bacteria for some antibiotics and this can impact susceptibility interpretation of clinical isolates.^11^ EUCAST breakpoints are dose-specific and use the EMA-approved doses that are included in the Summary of Product Characteristics of an antibiotic. This means that by application of breakpoints for higher doses, the effect of using a raised dose on the clinical efficacy of a particular antibiotic can be predicted. Currently, most clinical microbiology laboratories in India use CLSI breakpoints, however the international application of the EUCAST breakpoints is expanding,^12^ so it is possible that dose-specific breakpoints could at some time also be applied in India. The EUCAST dose-specific breakpoints can also be used retrospectively to calculate the susceptibility of previously collected isolates, to show the susceptibility levels that would have been achieved at higher doses.

The use of the EUCAST dose-specific breakpoints shows the effect of increasing the antibiotic dose on the susceptibility of a pathogen, providing additional information so the prescriber can decide if a higher dose would be of benefit. For example, *S. pneumoniae,* the most isolated respiratory pathogen^13,14^ from infections such as CAP, AOM and ABRS, has over time become less susceptible to amoxicillin/clavulanic acid in some countries^15^ since the MICs of some isolates have increased. When treating infections, it is important to be able to eradicate bacterial pathogens with raised MICs to optimize clinical outcome while at the same time minimizing the risk of selecting variants with even higher MICs. This is possible because β-lactams, unlike many other antibiotics, have time-dependent killing properties. Their efficacy depends on the amount of time the required drug concentration is present at the site of action. Although increasing the concentration at the infection site above a particular value will not have any effect on the efficacy, the use of higher doses and/or more frequent dosing allows for successful eradication of infections caused by pathogens with higher MICs because the time above the MIC at the site of infection is increased.^16^

**Figure 1.**
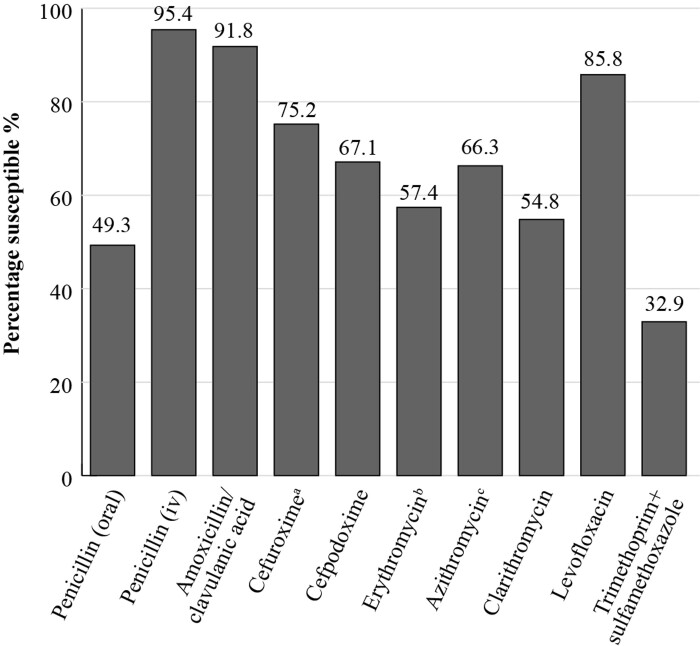
Percentage susceptibility rates based on CLSI breakpoints for antibiotics against *S. pneumoniae* isolates (*n *= 219) collected as part of the SOAR study in India in 2012–14. ^a^218 isolates tested. ^b^183 isolates tested. ^c^199 isolates tested.

**Figure 2.**
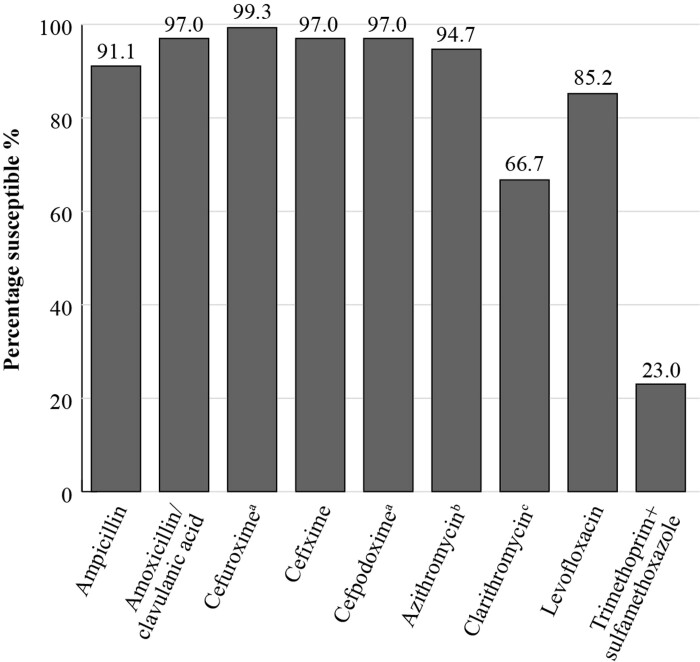
Percentage susceptibility rates based on CLSI breakpoints for antibiotics against *H. influenzae* isolates (*n *= 135) collected as part of the SOAR study in India in 2012–14. ^a^134 isolates tested. ^b^94 isolates tested. ^c^108 isolates tested.

SOAR results for India, collected from outpatients with confirmed CA-RTIs at four sites in 2012–14, show that when applying CLSI breakpoints, 91.8% of *S. pneumoniae* (*n *= 219) isolates were susceptible to amoxicillin/clavulanic acid, but this fell to 66.3% and 54.8% for the macrolides azithromycin and clarithromycin, respectively. For the fluoroquinolone, levofloxacin, *S. pneumoniae* susceptibility was 85.8%^10^ (Figure 1). For *H. influenzae* isolates (*n *= 135) from India, a high susceptibility of 97% to amoxicillin/clavulanic acid was seen, applying CLSI criteria. (Figure 2). Susceptibility to ampicillin was 91.1%, reflecting the presence of β-lactamase-positive isolates.^10^ The *H. influenzae* isolates showed a high susceptibility to the other antibiotics tested, except for clarithromycin (66.7% susceptible) and trimethoprim/sulfamethoxazole (23.0% susceptible).

#### ATLAS

**Figure 3.**
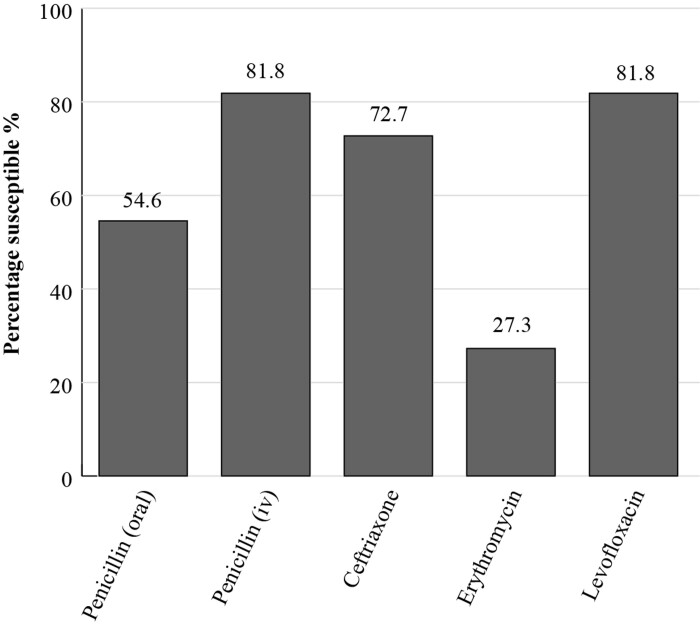
Percentage susceptibility rates based on CLSI breakpoints for antibiotics against *S. pneumoniae* isolates (*n *= 11) from the ATLAS surveillance programme in India in 2019. Data access date 11 November 2021.

**Figure 4.**
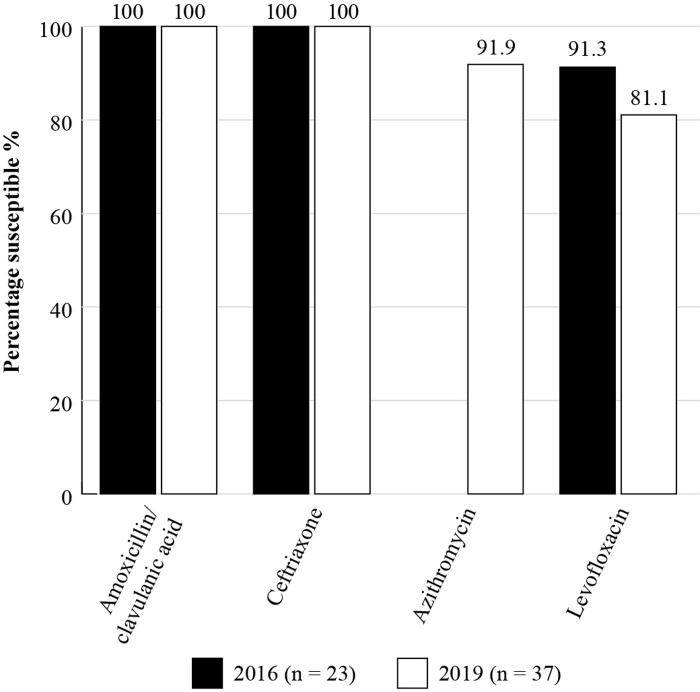
Percentage susceptibility rates based on CLSI breakpoints for antibiotics against *H. influenzae* isolates from the ATLAS surveillance programme in India in 2016 and 2019. Data access date 21 November 2021.

The Antimicrobial Testing Leadership and Surveillance (ATLAS) database is a global AMR surveillance programme which is fully searchable. It is available for general access and covers susceptibilities of a range of bacterial and fungal pathogens to various antimicrobials.^17^ ATLAS data is analysed based on CLSI and EUCAST breakpoints. Susceptibility data for India is currently available for only a small number of *S. pneumoniae* isolates (*n *= 11) from 2019 and *H. influenzae* isolates from 2016 (*n *= 23) and 2019 (*n *= 37) (Figures 3 and 4). *S. pneumoniae* was not tested against amoxicillin/clavulanic acid but the results available corroborate the reduction in susceptibility to macrolides (27.3%) as seen in the SOAR data. In the ATLAS data, *H. influenzae* isolates remained fully susceptible to amoxicillin/clavulanic acid and ceftriaxone in 2016 and 2019.^17^

#### GLASS

In 2015, WHO launched the Global Antimicrobial Resistance and Use Surveillance System (GLASS). GLASS is the first global system to collect national AMR data for selected bacterial pathogens that cause common infections. The aim is to monitor the prevalence of AMR among major pathogens in clinical settings to provide the supporting data required to ensure that countries can design cost-effective, evidence-based AMR response strategies.^18,19^ During the first 4 years, 91 countries or territories enrolled in GLASS and data for over two million patients from 66 countries are included.^19^ Pathogens currently included in GLASS-AMR are: *Acinetobacter* spp., *Escherichia coli*, *Klebsiella pneumoniae*, *Neisseria gonorrhoeae*, *Salmonella* spp., *Shigella* spp., *Staphylococcus aureus*, and *S. pneumoniae* and a new and important component is the inclusion of antimicrobial consumption (AMC) surveillance at the national level.^20^ GLASS data is analysed based on CLSI and EUCAST breakpoints. India is participating in the GLASS initiative but, according to the early implementation report for 2020, whilst *S. pneumoniae* isolates from blood samples will be included, no results are currently available within the system.^19^

## Disease Management Guidelines

For management of the common RTIs, CAP, AOM and ABRS, in India clinicians make use of a range of national/international antibiotic prescribing guidelines, examples of which are shown in Table 1. Most guidelines suggest a first-line antibiotic or antibiotics along with alternatives, and then a second-line antibiotic or antibiotics, also with alternatives. The first-line antibiotic is the recommended initial choice that should be prescribed by the clinician following diagnosis of the infection, supported by the criteria defined by the organization or committee; alternatives may be provided for use in particular circumstances. The second-line antibiotic is for use if the first-line antibiotic does not achieve the anticipated outcome, and again alternatives may be included for specific circumstances.

**Table 1.** Examples of local and international antibiotic prescribing guidelines referred to by physicians in India for the management of community-acquired respiratory tract infections

Local antibiotic prescribing guidelines
Joint Indian Chest Society (ICS) and the National College of Chest Physicians (NCCP) of India 2012: Guidelines for Diagnosis and Management of Community and Hospital-acquired Pneumonia in Adults^21^
National Centre for Disease Control (NTG) 2016: National Treatment Guidelines for Antimicrobial Use in Infectious Diseases^22^
Indian Council of Medical Research (ICMR) 2017: Indian Council of Medical Research Treatment Guidelines for Antimicrobial Use in Common Syndromes^23^
Indian Council of Medical Research (ICMR) 2019: Indian Council of Medical Research. Treatment Guidelines for Antimicrobial Use in Common Syndromes^24^
International antibiotic prescribing guidelines
IDSA 2007: Infectious Diseases Society of America. Guidelines on the Management of Community-acquired Pneumonia in Adults^25^
BTS 2009: British Thoracic Society Guidelines for the Management of Community-acquired Pneumonia in Adults: update 2009^26^
BTS 2011: British Thoracic Society Guidelines for the Management of Community-acquired Pneumonia in Children: update 2011^27^
IDSA/PIDS 2011: The Management of Community-Acquired Pneumonia in Infants and Children Older Than 3 Months of Age: Clinical Practice Guidelines by the Pediatric Infectious Diseases Society and the Infectious Diseases Society of America^28^
IDSA 2012: IDSA Clinical Practice Guideline for Acute Bacterial Rhinosinusitis in Children and Adults^29^
AAP 2013: American Academy of Pediatrics. The Diagnosis and Management of Acute Otitis Media^30^
AAP 2013: American Academy of Pediatrics: Clinical Practice Guideline for the Diagnosis and Management of Acute Bacterial Sinusitis in Children Aged 1 to 18 years^31^
NICE 2014: Pneumonia in Adults: Diagnosis and Management^32^
NICE 2017: Sinusitis (acute) Antimicrobial Prescribing^33^
NICE 2018: Otitis media (acute): Antimicrobial Prescribing^34^
NICE 2019: Pneumonia (community-acquired): Antimicrobial Prescribing^35^
IDSA 2019: Diagnosis and Treatment of Adults with Community-acquired Pneumonia. An Official Clinical Practice Guideline of the American Thoracic Society and Infectious Diseases Society of America^36^

### International antibiotic prescribing guidelines

For the management of CAP in adults and paediatrics, the international guidelines referred to by clinicians in India include those from the British Thoracic Society (BTS)^26,27^ the IDSA^28,36^ and from the UK’s National Institute for Health and Care Excellence (NICE).^35^ For example, a first-line antibiotic treatment recommendation for CAP management from the IDSA 2019 guideline for treating adults with no comorbidities or risk factors for MRSA or *Pseudomonas aeruginosa* is amoxicillin or doxycycline or a macrolide (if the local pneumococcal resistance is <25%) but if the patient has comorbidities, the recommendation is combination therapy with amoxicillin/clavulanic acid or a cephalosporin plus a macrolide or doxycycline, or monotherapy with a respiratory fluoroquinolone. The amoxicillin/clavulanic acid dose would be 875 mg/125 mg twice daily or 500 mg/125 mg three times daily or amoxicillin/clavulanic acid 2000 mg/125 mg twice daily plus either a macrolide or doxycycline^36^ and the NICE 2019 guidelines recommend amoxicillin/clavulanic acid for young people over 1 month and under 18 years and for adults as first-line treatment for severe symptoms.^35^ For the management of AOM, the international guidelines referred to in India include those from the American Academy of Pediatrics^30^ and NICE guidance.^34^ For the management of ABRS in adults and paediatrics, international guidelines used include those from the IDSA^29^ and NICE.^33^ For ABRS in children (with no penicillin allergy), the IDSA recommend initial empirical treatment as first-line, amoxicillin/clavulanic acid 45 mg/kg/day twice daily and in adults (with no penicillin allergy) first-line amoxicillin/clavulanic acid 500 mg/125 mg three times daily or 875 mg/125 mg twice daily.^29^

### National antibiotic prescribing guidelines

Concerning local Indian guidelines for management of CAP, AOM and ABRS, currently the commonly referred to documents are National Treatment Guidelines (NTG) for Antimicrobial Use in Infectious Diseases 2016,^22^ Treatment Guidelines for Antimicrobial Use in Common Syndromes 2019,^23^ and Joint ICS/NCCP(I) 2012 Guidelines for Diagnosis and Management of Community and Hospital-acquired Pneumonia in Adults.^21^ For example, the NTG 2016 National Treatment Guidelines for Antimicrobial Use in Infectious Diseases recommends amoxicillin/clavulanic acid for the treatment of ABRS in adults at a dose of 1 g/day twice daily and for severe ABRS in children, 45 mg/kg/day twice daily.^22^

## Antibiotic availability

Access to antibiotics may be an issue for patients in low- and middle-income countries. Drug supply chains may also contribute to the problem. Limited access to the most appropriate antibiotic to treat a specific infection may result in increased mortality from treatable bacterial infections and, the use of suboptimal amounts of antibiotic facilitates resistance development and allows resistant strains to persist.^37,38^

In India, taking amoxicillin/clavulanic acid as an example, several currently available formulations are mentioned as first-line or second-line recommendations by the RTI management guidelines. Examples include combination therapy of amoxicillin/clavulanic acid 500 mg/125 mg three times daily with a macrolide, which is recommended in the NICE 2019 guidelines for the treatment of CAP in adults and children with severe CAP. In the ICMR 2019 guidelines for adult CAP patients with no comorbidities, amoxicillin/clavulanic acid 1 g twice daily or 625 mg three times daily is recommended. It was not until April 2021 that amoxicillin/clavulanic acid 90 mg/kg or 4 g/0.25 g per day (high-dose) was available in India. High-dose regimens are recommended by: IDSA 2007 CAP treatment guidelines^25^ for outpatients with comorbidities; IDSA 2011 guidelines for the treatment of paediatric CAP in outpatients;^28^ IDSA 2012 treatment guidelines for sinusitis^29^ as initial treatment for children and in adults with risk of treatment failure; AAP 2013 guidelines as initial or delayed treatment of AOM in children^30^ or after failure of initial treatment; and by the IDSA 2019 guidelines for adults CAP patients with comorbidities.^36^

Substandard, poor-quality or falsified antibiotics promote AMR and the spread of drug-resistant infections,^39^ since poor-quality antibiotics are unlikely to contain the full dose needed to eliminate all of the infecting pathogens, which will encourage resistance to develop and allow resistant strains to survive and be transmitted.^40^ The quality of medicines, specifically antibiotics, is an important consideration for countries worldwide. The WHO launched a Global Surveillance and Monitoring System (GSMS) for substandard and falsified products.^40^ The GSMS aims to work with WHO member states to improve the quality of reporting of substandard and falsified medical products, and, importantly, to ensure the data collected are analysed and used to influence policy, procedure, and processes to protect public health, at the national, regional and the global level. Use of substandard or falsified antibiotics not only compromises clinical outcome but also risks increased AMR. The most recent summary (2013–17) reported substandard and falsified medicines in 46 member states (including India) and antibiotics represent 16.9% of all products reported, second only to malaria drugs (19.6%).^40^

## Local insights

### Clinical microbiologist expert comment

From a clinical microbiologist’s perspective, AST, performed for clinically relevant bacterial isolates based on the site of infection is vital, not only for guiding therapy for a specific patient but also for understanding epidemiological trends of different pathogens and for the choice of antimicrobials used for empirical or targeted therapy. Every healthcare facility should maintain its local antibiogram for important pathogens due to variation between areas, as illustrated by studies of *S. pneumoniae* in India which reveal a range in penicillin resistance from 3.8% to 30%.^41^ In such a large country as India, it is important to capture susceptibility variation in order to optimize antibiotic use.

Antibiograms, along with clinical outcome data, contribute to the preparation of local, national and international guidelines for empirical antibiotic prescribing. Without local or national data, one can still refer to international guidelines especially for community-acquired pathogens to define empirical choices. Antibiotic prescribing guidelines allow for standardization of therapy with respect to primary and secondary choices, route, dose, frequency and optimum duration for treatment. In the absence of such guidelines, antibiotic abuse prevails which further drives AMR. Another factor driving antibiotic use is access. In India, access to antibiotics is a major cause of concern because of the extensive availability of OTC antibiotics. It has been suggested that in some situations, when there is limited access to a healthcare professional (HCP) or in resource-limited settings, OTC purchase of antibiotics may be needed, but self-medication risks antibiotic misuse and can result in increasing resistance.^42^ Further risk factors for AMR include counterfeit medications and sharing antibiotics between family members. Balancing access and excess therefore remains key to minimizing antibiotic abuse and AMR. Primary care and outpatient settings need to adopt AMS, diagnostic stewardship (making diagnostics readily available) and infection prevention and control practices to use antibiotics effectively. Capacity building of community health workers, pharmacists and doctors towards appropriate antibiotic use and improving community involvement through education and awareness will minimize antibiotic abuse in the long term. The success of any of these interventions requires strong political will and leadership support.

Common challenges faced by clinical microbiology laboratories when undertaking and reporting the results of AST include inappropriate specimen provision (e.g. sending swabs instead of fluids or tissue samples), AST reports being requested: (i) for colonizers; (ii) when there is ‘no growth’; (iii) for antibiotics that do not concentrate at the infection site; (iv) for antibiotics that are not active against the pathogen; and (v) for antibiotics that are not included in CLSI/EUCAST or local guidelines. In addition, physicians may compare and prefer non-standard reporting patterns (+/−) with standard reporting (S/I/R) as per CLSI or EUCAST. There is also a lack of understanding among clinicians on how to use MICs, especially for drug-resistant pathogens. Lack of patient histories may result in physicians requesting AST reports for whatever has grown, which overestimates infection rates, especially at non-sterile sites, leading to unnecessary antibiotic prescribing. Poor communication between clinical microbiologists and physicians may result from lack of insight into each other’s field of expertise.

Frequent informal and formal discussion between both experts on challenging cases or samples that need to be sent for appropriate diagnosis, antibiotics to be tested, mechanisms of resistance, and escalation or de-escalation strategies would greatly help. Good IT back up is needed for quick and sustained communication of AST results and hospital-based laboratories should ensure continuous availability of microbiology services along with written guidance to the clinical team on specimen collection, storage and transport, in order to optimize recovery of pathogens. Regular clinicopathological co-relation meetings/journal clubs to discuss cases from the clinical and microbiology perspectives would promote communication and understanding along with participation of microbiologists in clinical rounds to open channels for bedside case discussions and exchange of information. Inclusion of advisory notes on AST reports regarding preferred antibiotic choice, mechanism of drug resistance, additional tests that may support clinical diagnosis or management of the infectious disease would also improve the situation. There is also an urgent need to introduce compulsory refresher training programmes for all HCPs prescribing antimicrobial agents at intervals to understand and adopt good antimicrobial prescribing practices. Finally, annual, or more frequent, discussions between different specialities regarding antibiotic patterns of important pathogens and empirical choices of therapy to formulate the antibiotic policy would be beneficial.

### Clinician expert comment

From a clinician’s perspective in India, recent local susceptibility data would assist in the selection of empirical antibiotics for community-acquired infection management and to support rational choices when treating these bacterial infections. In addition, HCPs should be regularly alerted to rational antibiotic use. A community-acquired pathogen susceptibility registry that is India-specific is lacking; only sporadic data are available in published papers. There are many good initiatives underway from the Indian government, such as the ICMR, but the data published here are mainly related to Gram-negative hospital-acquired infections, which is not helpful to the physician managing CA-RTIs. In addition, antibiograms produced by major hospitals do not reflect the situation within the community, however if they do exist for a specific hospital, they should be updated on an annual basis.

In clinical practice in India, it is advisable to check any available local antibiotic surveillance data that exists ahead of prescribing antibiotics, along with local management guidelines including those produced by the ICMR, Indian Academy of Pediatrics (IAP) and NTG, and to check pathogen prevalence data, management guidelines and local hospital susceptibility data, if available. Patient education is also vital, covering the misuse of antibiotics, such as not purchasing OTC antibiotics and also the need to complete the full course as prescribed by the physician. This is where campaigns, such as ‘Medicines with the Red Line’, which educate the public and help enforce pharmacy laws can be effective but need to be regularly revisited and refreshed. AMS programmes are also important, and they should be implemented at all levels, including in the pharmacy. Most patients do not complete the full course of antibiotics as prescribed by HCPs. Parents should also be educated about the need for, and the importance of, completing the course of antibiotics as prescribed. There should be regular continuing medical education (CME) and awareness programmes for HCPs about rational selection and appropriate use of antibiotics.

## Conclusions

In an era of rising AMR throughout the world, this paper aims to define areas where action is required to address AMR by analysing and understanding the current situation within India. Information is presented concerning antibiotic use and prescribing, approach to AMR, availability of local susceptibility data, use of international and/or local management guidelines and how these link to antibiotic availability. To our knowledge, this is the first time this information has been reviewed and presented in detail by country.

Antibiotic use in India is extremely high; in 2010, India was the world’s largest consumer of antibiotics for human health and the burden of infectious diseases in India is amongst the highest in the world. In terms of surveillance, there are a small number of studies from individual units or hospitals that have provided information on local AMR and the ICMR have established an AMR surveillance network (ICMR-AMRSN) in tertiary care hospitals across India. Several global surveillance studies also include data for India including SOAR and ATLAS. The WHO GLASS study will also provide useful data in the future, but complete and robust surveillance data is required for bacterial pathogens responsible for community-acquired infections.

For management of the common RTIs, CAP, AOM and ABRS in India, clinicians make use of several country-specific local antibiotic prescribing guidelines plus a wide range of international antibiotic prescribing guidelines. For the management of CA-RTIs, amoxicillin/clavulanic acid is an antibiotic commonly recommended by both international and local prescribing guidelines. Susceptibility studies (ATLAS and SOAR) confirm that susceptibility of the common RTI pathogens, *S. pneumoniae* and *H. influenzae* remains high, supporting these recommendations in India.

The clinical microbiologist and clinician are united in their wish for local up-to-date susceptibility data to support empirical antibiotic prescribing. An increase in local surveillance studies would allow local management guidelines to be updated regularly which, if applied along with guidelines relating to the management of viral diseases such as COVID-19 would improve clinical outcomes and minimize further rises in AMR. While a range of international guidelines is utilized by clinicians in India, a more standardized inclusive approach is needed to develop local country-specific guidelines. These guidelines would be based on up-to-date surveillance data of isolates from community-acquired infections, which would make them more locally relevant for clinicians, reiterating the Consensus Principles as described in the introductory paper to this Supplement.^2^ This would pave the way for improved adherence and a higher level of appropriate antibiotic prescribing in CA-RTIs which could, in turn, potentially limit AMR development and improve clinical outcomes for patients.

## References

[1] WHO . Antibiotic resistance – fact sheet. 2020. https://www.who.int/news-room/fact-sheets/detail/antibiotic-resistance.

[2] Cantón R , Akova M, Langfeld K et al Relevance of the Consensus Principles for Appropriate Antibiotic Prescribing in 2022. J Antimicrobial Chemother 2022; 77 Suppl 1: dkac211. 10.1093/jac/dkac211 PMC9445850 36065724

[3] WHO . Antimicrobial resistance – fact sheet. 2021. https://www.who.int/news-room/fact-sheets/detail/antimicrobial-resistance.

[4] Ranjalkar J , Chandy SJ. India's National Action Plan for antimicrobial resistance - an overview of the context, status and way ahead. J Family Med Prim Care 2019; 8: 1828–1834. 31334140 10.4103/jfmpc.jfmpc_275_19 PMC6618210

[5] WHO . Global action plan on antimicrobial resistance. 2015. https://www.who.int/publications/i/item/9789241509763.

[6] Lahiry S , Ramalingam R, Dalal K et al Tackling AMR crisis in India: changing paradigm. Asian J Med Sci 2020; 11: 129–37.

[7] Worldometer . India Population. 2022. https://www.worldometers.info/world-population/india-population/.

[8] Ministry of Health and Family Welfare, Government of India . National Action Plan in antimicrobial resistance. 2020. https://rr-asia.oie.int/wp-content/uploads/2020/03/india_national-action-plan-on-amr-india.pdf.

[9] Laxminarayan R , Duse A, Wattal C et al Antibiotic resistance—the need for global solutions. Lancet Infect Dis 2013; 13: 1057–98. 24252483 10.1016/S1473-3099(13)70318-9

[10] Torumkuney D , Chaiwarith R, Reechaipichitkul W et al Results from the Survey of Antibiotic Resistance (SOAR) 2012–14 in Thailand, India, South Korea and Singapore. J Antimicrob Chemother 2016; 71 Suppl 1: i3–19. 27048580 10.1093/jac/dkw073 PMC4890353

[11] Turnidge J , Paterson DL. Setting and revising antibacterial susceptibility breakpoints. Clin Microbiol Rev 2007; 20: 391–408. 17630331 10.1128/CMR.00047-06 PMC1932754

[12] EUCAST . Implementation of EUCAST breakpoints/guidelines. 2022. https://www.eucast.org/fileadmin/src/media/PDFs/EUCAST_files/Statistics/EUCAST_Maps_March_2022.pdf.

[13] Welte T , Torres A, Nathwani D et al Clinical and economic burden of community-acquired pneumonia among adults in Europe. Thorax 2010; 67: 71–9. 20729232 10.1136/thx.2009.129502

[14] Jain S , Self WH, Wunderink RG et al Community-acquired pneumonia requiring hospitalization among U. S. adults. N Engl J Med 2015; 373: 415–27. 26172429 10.1056/NEJMoa1500245 PMC4728150

[15] Torumkuney D , Gur D, Soyletir G et al Results from the Survey of Antibiotic Resistance (SOAR) 2002–09 in Turkey. J Antimicrob Chemother 2016; 71 Suppl 1: i85–91. 27048585 10.1093/jac/dkw067 PMC4890349

[16] Jacobs MR . Building in efficacy: developing solutions to combat drug-resistant *S. pneumoniae*. Clin Microbiol Infect 2004; 10 Suppl 2: 18–27. 10.1111/j.1470-9465.2004.00862.x 14759230

[17] Pfizer . Antimicrobial surveillance. https://www.pfizer.com/science/therapeutic-areas/anti-infectives/antimicrobial-surveillance.

[18] Inoue H . Strategic approach for combating antimicrobial resistance (AMR). Glob Health Med 2019; 1: 61–4. 33330756 10.35772/ghm.2019.01026 PMC7731180

[19] WHO . Global Antimicrobial Resistance and Use Surveillance System (GLASS) Report. Early implementation. 2020. https://apps.who.int/iris/bitstream/handle/10665/332081/9789240005587-eng.pdf.

[20] WHO . Global Antimicrobial Resistance and Use Surveillance System (GLASS) Report: 2021. 2021. https://www.who.int/publications/i/item/9789240027336.

[21] Gupta D , Agarwal R, Nath Aggarwal A et al Guidelines for diagnosis and management of community- and hospital-acquired pneumonia in adults: Joint ICS/NCCP(I) recommendations. Lung India 2012; 29: S27–62. 23019384 10.4103/0970-2113.99248 PMC3458782

[22] National Centre for Disease Control . National Treatment Guidelines for Antimicrobial Use in Infectious Diseases. 2016. https://ncdc.gov.in/WriteReadData/l892s/File622.pdf.

[23] Indian Council of Medical Research . Treatment Guidelines for antimicrobial use in common syndromes. 2017. https://main.icmr.nic.in/sites/default/files/guidelines/Treatment_guidelines_2017.pdf.

[24] Indian Council of Medical Research . Treatment Guidelines for antimicrobial use in common syndromes. 2019. https://icmr.org.in/images/pool/guidelines/Treatment_Guidelines_2019_Final.pdf.

[25] Mandell LA , Wunderink RG, Anzueto A et al Infectious Diseases Society of America/American Thoracic Society consensus guidelines on the management of community-acquired pneumonia in adults. Clin Infect Dis 2007; 44: S27–72. 17278083 10.1086/511159 PMC7107997

[26] Lim WS , Baudouin SV, George RC et al BTS guidelines for the management of community acquired pneumonia in adults: update 2009. Thorax 2009; 64 Suppl 3: iii1–55. 19783532 10.1136/thx.2009.121434

[27] Harris M , Clark J, Coote N et al British Thoracic Society guidelines for the management of community acquired pneumonia in children: update 2011. Thorax 2011; 66 Suppl 2: ii1–23. 21903691 10.1136/thoraxjnl-2011-200598

[28] Bradley JS , Byington CL, Shah SS et al The management of community-acquired pneumonia in infants and children older than 3 months of age: clinical practice guidelines by the Pediatric Infectious Diseases Society and the Infectious Diseases Society of America. Clin Infect Dis 2011; 53: e25–76. 21880587 10.1093/cid/cir531 PMC7107838

[29] Chow AW , Benninger MS, Brook I et al IDSA clinical practice guideline for acute bacterial rhinosinusitis in children and adults. Clin Infect Dis 2012; 54: e72–112. 22438350 10.1093/cid/cir1043

[30] Lieberthal AS , Carroll AE, Chonmaitree T et al The diagnosis and management of acute otitis media. Pediatrics 2013; 131: e964–99. 23439909 10.1542/peds.2012-3488

[31] Wald ER , Applegate KE, Bordley C et al Clinical practice guideline for the diagnosis and management of acute bacterial sinusitis in children aged 1 to 18 Years. Pediatrics 2013; 132: e262–80. 23796742 10.1542/peds.2013-1071

[32] NICE . Guideline cg191 Pneumonia in adults: diagnosis and management [Guidance withdrawn by NICE and currently under review]. 2014. https://www.nice.org.uk/guidance/cg191.

[33] NICE . Guideline ng179. Sinusitis (acute): antimicrobial prescribing. https://www.nice.org.uk/guidance/ng179.

[34] NICE . Guideline ng91. Otitis media (acute): antimicrobial prescribing. https://www.nice.org.uk/guidance/ng91.

[35] NICE . Guideline ng138. Pneumonia (community-acquired): antimicrobial prescribing. https://www.nice.org.uk/guidance/ng138.

[36] Metlay JP , Waterer GW, Long AC et al Diagnosis and treatment of adults with community-acquired pneumonia. An Official Clinical Practice Guideline of the American Thoracic Society and Infectious Diseases Society of America. Am J Respir Crit Care Med 2019; 200: e45–67. 31573350 10.1164/rccm.201908-1581ST PMC6812437

[37] Ball P , Baquero F, Cars O et al Antibiotic therapy of community respiratory tract infections: strategies for optimal outcomes and minimized resistance emergence. J Antimicrob Chemother 2002; 49: 31–40. 10.1093/jac/49.1.31 11751764

[38] Centre for disease dynamics, economics and policy . Access barriers to antibiotics. 2019. https://cddep.org/wp-content/uploads/2019/04/access-barriers-to-antibiotics.pdf.

[39] Nwokike J , Clarke A, Nguyen PP. Medicines quality assurance to fight antimicrobial resistance. Bull World Health Organ 2018; 96: 135–7. 29403117 10.2471/BLT.17.199562 PMC5791778

[40] WHO . Substandard and falsified medical products. https://www.who.int/health-topics/substandard-and-falsified-medical-products#tab=tab_1.

[41] Peela MSR , Sistla S, Tamilarasu K et al Antimicrobial resistance in clinical isolates of *Streptococcus pneumoniae:* mechanisms and association with serotype patterns. J Clin Diagn Res 2018; 12: DC17–21.

[42] Morgan DJ , Okeke IN, Laxminarayan R et al Non-prescription antimicrobial use worldwide: a systematic review. Lancet Infect Dis 2011; 11: 692–701. 21659004 10.1016/S1473-3099(11)70054-8 PMC3543997

